# Role of Ubiquitin-Specific Peptidase 47 in Cancers and Other Diseases

**DOI:** 10.3389/fcell.2021.726632

**Published:** 2021-09-17

**Authors:** Kailing Pan, Junhao Fu, Wenxia Xu

**Affiliations:** Central Laboratory, Affiliated Jinhua Hospital, Zhejiang University School of Medicine, Jinhua, China

**Keywords:** deubiquitinase, USP47, cancer, ubiquitination, USP7

## Abstract

Deubiquitination is the reverse process of ubiquitination, which is catalyzed by deubiquitinase enzymes. More than 100 deubiquitinases have been identified. Ubiquitin-specific peptidase 47 (USP47), a member of the ubiquitin-specific protease family with high homology to USP7, is an active molecule with a wide range of functions and is closely associated with cancer and other diseases. However, no systematic summary exists regarding the functions of USP47. Here, we summarize the functions and expression regulation of USP47. USP47 is highly expressed in many tumors and is widely involved in tumor development, metastasis, drug resistance, epithelial-mesenchymal transition, and other processes. Targeted inhibition of USP47 can reverse malignant tumor behavior. USP47 also plays a role in inflammatory responses, myocardial infarction, and neuronal development. USP47 is involved in multiple levels of expression-regulating mechanisms, including transcriptional, post-transcriptional, and post-translational modifications. Development of targeted inhibitors against USP47 will provide a basis for studying the mechanisms of USP47 and developing therapeutic strategies for cancers and other diseases.

## Introduction

Proteins are the executors of life. Intracellular protein quality must be strictly and precisely controlled to perform various physiological functions. Protein quality control depends on the balance between synthesis and decomposition. Protein synthesis undergoes DNA transcription and RNA translation, which is known as the Central Dogma (Cooper, 1981). Protein degradation depends on three pathways: the ubiquitin-proteasome pathway, autophagy pathway, and proteolysis pathway (Beynon and Bond, 1986; Blommaart et al., 1997; Komander and Rape, 2012). Incorrect synthesis and abnormal expression of proteins under various stress conditions triggers the ubiquitin-proteasome pathway in cells to maintain homeostasis. Approximately 80% of cellular proteins are degraded *via* this pathway (Amm et al., 2014). Abnormalities in the ubiquitin-proteasome pathway can lead to the development of many diseases, including cancer (Mani and Gelmann, 2005; Bijlmakers, 2020; Fhu and Ali, 2021; Schmidt et al., 2021).

Deubiquitination is the reverse process of ubiquitination, which is catalyzed by deubiquitinase (DUB) enzymes. Deubiquitinase hydrolyzes the ubiquitin molecule from the ubiquitinated protein by hydrolyzing the ester bond, peptide bond, or isopeptide bond at the carboxyl terminal of the ubiquitin molecule and reverses the ubiquitination level of the cellular protein to maintain the protein’s stability (Amerik and Hochstrasser, 2004; Ritorto et al., 2014). Deubiquitinase also inhibits the ligation function of E3 ligase, mainly by cutting the peptide bond between the lysine residue on the target protein and the C-terminus of ubiquitin, affecting the substrate and ubiquitin chain to achieve deubiquitination (D’Arcy et al., 2015). Many types and quantities of deubiquitination enzymes exist owing to the large number of substrate proteins. More than 100 deubiquitinases have been identified and can be divided into either cysteine proteinases or metalloproteinases according to the active site. Cysteine proteases include the ubiquitin-specific protease (USP) family, ubiquitin-C-terminal hydrolase (UCH) family, Machado-Josephin domain protease (MJD) family, and ovarian tumor-associated protease (OTU) family. Metalloproteases mainly belong to the MPN (+)/JAMM protease family (Pinto-Fernández et al., 2019). Of these families, the USP family has the most members (more than 50), making it the most studied deubiquitinase family. These members are widely involved in cancer, inflammation, immunity, and neurological diseases.

Ubiquitin-specific peptidase 47 (USP47) is a member of the USP family with high homology with USP7. USP47 processes Lys48- and Lys63-linked polyubiquitin chains into monomeric ubiquitin time-dependently, and more efficiently than does USP7 (Piao et al., 2015). The human USP47 gene (120,465 bases) is located on chromosome 11p15.3 and shares a similarity of 99.73, 89.54, and 89.3% to the chimpanzee, mouse, and rat USP47 genes, respectively.^1^ The USP47 gene encodes a protein of 1,375 amino acids (aa), with an approximate molecular weight of 157 kDa (Piao et al., 2015). It has two additional transcripts of the USP47 gene, which produce two proteins of 1,287aa (∼147 kDa) and 1,355aa (∼155 kDa), respectively.^2^ Gene ontology annotations of all three transcripts include thiol-dependent USP activity and cysteine-type peptidase activity. According to the Human Protein Atlas, USP47 is mainly found in the intermediate filaments and vesicles. USP47 is widely expressed in most organs, including the brain, liver, and kidneys, thus suggesting that USP47 plays a vital role in regulating the life activities of organisms. USP47 is an active molecule with a wide range of functions and is closely related to cancers and other diseases, including neurological diseases and inflammatory responses. However, no summary exists of the functions of USP47. In this mini review, we summarize the functions and expression regulation of USP47.

## Structure of Ubiquitin-Specific Peptidase 47

**FIGURE 1:**
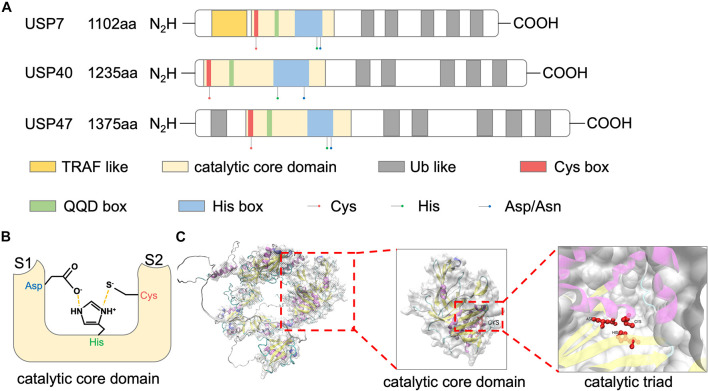
Structure of USP47. **(A)** Schematic representations of domain architectures of USP7, USP40, and USP47. **(B)** Schematic overview of the catalytic triad of USP47 constructed by Cys, His, and Asp. Nearby S1 and S2 sites accommodate the proximal and distal ubiquitin units, respectively. **(C)** The tertiary structure of USP47 predicted by AlphaFold prediction (https://alphafold.ebi.ac.uk/entry/Q96K76) and visualized by VMD.

Ubiquitin-specific peptidase 47 is a cysteine protease with three isoforms in human, including a full-length transcript of 1,375aa and two N-terminal 14–101 and 14–33 deleted transcripts. Among the USP family, USP47 shows high sequence similarity to USP7 and USP40. These proteins share a common structural feature, including an N-terminal catalytic core domain and multiple ubiquitin-like domains (UBLs) (Figure 1A; Clague et al., 2013).

### Catalytic Core Domain

The largest USP47 domain is the N-terminal catalytic core, spanning residues 188–564 (Clague et al., 2013). The catalytic activity of USP47 relies on a conserved catalytic triad composed of Cys, His, and Asp/Asn residues. The N-terminal of this domain is a “Cys box,” containing a catalytic cysteine residue. Replacement of the catalytic cysteine with a serine residue impaired the deubiquitination activity of USP47 (Shi et al., 2015; Yu et al., 2019; Pan et al., 2020). Following this “Cys box” are non-catalytic residues of the “QQD box.” The second part of the catalytic triad is a histidine residue located in the “His box” which is behind the QQD box. The third and the last component of the catalytic triad is an aspartic acid. Several residues followed the C-terminus of this histidine (Quesada et al., 2004). As shown in Figure 1B, the histidine assists the deprotonation of the catalytic cysteine and promotes a nucleophilic attack on the carbonyl carbon atom of the ubiquitin, while the aspartic acid is used to stabilize the histidine (Clague et al., 2013). The predicted tertiary structure of USP47 is shown in Figure 1C and Jumper et al. (2021). Besides the catalytic property, studies have shown that the catalytic domain also plays a role in protein–protein interactions. Deleting this domain abolished the interaction of USP47 and multiple proteins, including β-TrCP, SATB1, and YAP (Shi et al., 2015; Yu et al., 2019; Pan et al., 2020). The study by Shi et al. (2015) investigating USP47 and β-TrCP interactions found that, unlike the typical β-TrCP binding site (DSGxxS), USP47 presents a novel recognition motif (DEGxxxE), in which the glutamic acid could mimic a phosphoserine. This indicated that the catalytic core domain of USP47 might also be involved in mediating protein–protein interactions, and its binding motif may be decided by the target proteins.

### Ubiquitin-Like Domains

As USP40 and USP7, USP47 has multiple UBL domains in addition to the catalytic core domain. Many studies aimed to clarify the functions of the USP7 UBL domains. Firstly, it was revealed that the UBLs at the C-terminus of USP7 could increase the catalytic activity by promoting the rearrangement of the catalytic triad (Faesen et al., 2011; Piao et al., 2015). Secondly, USP47 UBLs can serve as a substrate-binding platform, in which the “RxxKxxxK” motif of UBL and the “KxxxKxK” motif of the substrate can be used to predict binding to the UBL domains (Pfoh et al., 2015; Kim and Sixma, 2017). As for USP47, the deficiency of UBLs impaired the binding between USP47 and RPS2, indicating that the UBLs of USP47 were also involved in substrate binding (Cho et al., 2020).

To date, there have been no structural studies of USP47. Solution NMR studies on the structure of the catalytic core domain and UBLs will help to get a complete understanding of the USP47 function and accumulate the development of specific USP47 inhibitors.

## Physiological Functions of Ubiquitin-Specific Peptidase 47

The physiological functions of USP47 have not been extensively investigated. To date, reports have been published on the function of USP47 in axonal growth, cell adhesion, epithelial-mesenchymal transition (EMT), and DNA damage repair.

### Axonal Growth

It has been reported that USP47 promotes axonal growth in cultured rat hippocampal neurons. Mechanistically, USP47 deubiquitinates katanin, a heterodimeric enzyme that cleans and breaks down microtubules, playing an essential role in axonal growth. Both USP47 and CHIP, an E3 ubiquitin ligase, competitively bind to katanin’s AAA ATPase domain. USP47 antagonizes CHIP-mediated katanin degradation, therefore controlling axonal growth during neuronal development (Yang et al., 2013).

### Cell Adhesion

E-cadherin is a key molecule in the process of epithelial cell adhesion, and USP47 is essential for maintaining epithelial cell adhesion *via* E-cadherin deubiquitination. Studies showed that USP47 could be recruited to adhesive junctions *via* KIFC3, a minus end-directed motor. Hakai, an E3 ubiquitin protein ligase, is involved in the cleavage of E-cadherin. The E-cadherin cleavage produced a 90-kDa fragment, which is then further degraded by lysosomes, hence blocking cell adhesion. USP47 degrades Lys48-linked polyUb chains formed by Hakai in the cytoplasmic region of E-cadherin, protecting E-cadherin from polyUb chain-induced lysosomal degradation (Sako-Kubota et al., 2014).

### Epithelial-Mesenchymal Transition

Ubiquitin-specific peptidase 47 also helps to promote EMT in normal cells (Silvestrini et al., 2020). One study, using MCF-10A cells induced with TGFβ2 as an EMT model, found that USP47 was one of the top upregulated proteins by quantitative proteomics. Additionally, chemical inhibition of USP47 reduced the expressions of several EMT markers, including CDH1, CTNNB1, and SNAIL, and reversed the morphological changes in MCF-10A cells undergoing EMT (Silvestrini et al., 2020). E-cadherin is also an important molecule involved in EMT. As mentioned above, E-cadherin is deubiquitinated by USP47. However, the relationship between EMT mediated by USP47 and E-cadherin was not explored in this study. Hence, the role of USP47 in the EMT process and its regulatory mechanism still needs to be further elucidated.

### DNA Damage Repair

DNA damage is closely related to tumorigenesis. The DNA polymerase Polβ is a core molecule of the base excision repair pathway. Parsons et al. (2011) found that USP47 could deubiquitinate Polβ, and USP47 knockdown inhibited the base excised repair pathway and increased hydrogen peroxide-induced DNA damage sensitivity. Therefore, USP47 is necessary to regulate DNA repair and maintain genomic stability. Additionally, USP47 is indirectly involved in DNA damage responses by stabilizing the spliceosomal protein IK to complete the splicing of the ATM precursor mRNA (Ka et al., 2020). USP47-deficient MEF cells isolated from a USP47 knockout mouse model revealed that USP47 knockout significantly increased the levels of ultraviolet-induced apoptosis (Peschiaroli et al., 2010). These studies suggest that targeted USP47 may maintain the normal state of cells and block tumorigenesis.

## Role of Ubiquitin-Specific Peptidase 47 in Cancer

Ubiquitin-specific peptidase 47 plays a role in various cancers, including colorectal cancer (CRC), breast cancer, lung cancer, and gastric cancer.

### Colorectal Cancer

Colorectal cancer is the third most common cancer globally, and patients with metastatic stage IV CRC have a low overall 5-year survival rate (about 15%) (Bray et al., 2018). Therefore, early screening and diagnosis are critical to survival. A research using a gene expression omnibus (GEO) dataset, GDS2609, containing mRNA expression data of colon mucosae from early-onset CRC patients and healthy controls showed that USP47 mRNA levels were increased in CRC samples (Pan et al., 2020). Besides, studies in patients with CRC have shown that USP47 was expressed more abundantly in tumors than their normal counterparts, and a higher expression of USP47 was positively correlated with a higher probability of lymph node metastasis and predicted a shorter overall survival (Pan et al., 2020; Zhang et al., 2021). Moreover, immunohistochemical analysis of USP47 protein levels in a tissue microarray composed by 90 CRC samples found higher USP47 expression in tumor tissues, and USP47 expression was positively correlated with tumor size (Pan et al., 2020). These studies suggest that USP47 might be a potential biomarker and prognostic indicator for the diagnosis of CRC.

Ubiquitin-specific peptidase 47 has been shown to be involved in cell proliferation, cell survival, and cell migration at the cellular level. Mechanically, USP47 participated in the regulation of multiple signaling pathways, including Hippo, EMT, and P53 signaling pathways. YAP, which is frequently overexpressed in CRC, is an important effector of the Hippo pathway. USP47 served as a deubiquitinase for YAP. The USP47 peptidase and coiled-coil domains mediated the interaction with the YAP N-terminal domain and directly regulated YAP deubiquitination leading to its stabilization. Moreover, USP47 knockdown could repress CRC cell proliferation, which could be rescued by YAP overexpression (Pan et al., 2020). EMT is a process that is important in cancer metastasis due to its function in the re-organization of the cytoskeleton. USP47 has been shown to induce the decomposition of E-cadherin and promote the epithelial–mesenchymal transition *via* Snail-deubiquitination (Choi et al., 2017). This work was the first work to show that USP47 regulates the EMT and metastasis in CRC cells. SATB1 is a genome organizer that facilitates chromatin organization and regulates gene expression. USP47 interacted with SATB1 through its catalytic core domain and antagonized Smurf2-mediated SATB1 degradation. The deficiency of USP47 impaired the transcriptional activity of SATB1 target genes and inhibited cell proliferation, migration, and tumorigenesis in a mouse model of colon cancer. Besides, USP47 knockout could increase E-cadherin expression and inhibited β-catenin expression in HCT116 cells, which is the reverse process of EMT. These results suggested that USP47 could mediate EMT by stabilizing SATB1 (Yu et al., 2019). However, as mentioned above, E-cadherin can also be deubiquitinated and stabilized by USP47. Therefore, the modulation of E-cadherin by USP47 may be related to the cell states. In a normal state, USP47 binds to E-cadherin, preventing its degradation; however, when cells are under hypoxic conditions or during EMT, the binding of USP47 to Snail and SATB1 is enhanced, thereby inhibiting the expression of E-cadherin. In addition, studies showed that USP47 is involved in the regulation of colon cancer through P53. USP47 knockdown significantly inhibited cell proliferation and clone formation in HCT116 CRC with the P53 wild-type but not in P53 knockout HCT116 cells. Further mechanistic studies showed that USP47 deubiquitinated RPS2, thereby inhibiting interaction between RPS2 and MDM2, and alleviated RPS2-mediated MDM2 inhibition under normal conditions, thus inhibiting P53. USP47 knockdown led to an increased binding of RPS2 to MDM2, inhibiting MDM2 activity and upregulating p53 levels under ribosomal stress, thereby inhibiting cell proliferation, colony formation, and tumor progression (Cho et al., 2020). Another study found that USP47 promoted CRC development by maintaining the stemness of CRC stem cells (Zhang et al., 2021). Moreover, due to the vital role of USP47 in DNA damage repair, the mechanisms mediating CRC chemotherapy resistance deserve further investigation. Thus, USP47 may be a potential therapeutic target for CRC.

### Other Cancers

According to the Protein Atlas database, USP47 is expressed in several cancers. The highest USP47 expression was found in breast cancer. Higher USP47 expression was also associated with breast cancer metastasis and a lower survival probability (Silvestrini et al., 2020). Knockdown of USP47 expression in human osteosarcoma U-2OS and SAOS-2 cells and breast cancer T47D, BT-20, and MCF7 cells significantly induced apoptosis and improved the sensitivity to chemotherapeutic drugs.

Ubiquitin-specific peptidase 47 knockdown inhibited the proliferation of A549 lung cancer cells and PC3 prostate cancer cells by deubiquitinating β-catenin (Shi et al., 2015). Interfering with USP47 expression using siRNA increased the ubiquitination and resultant degradation of β-catenin, inhibiting the Wnt signaling pathway. Furthermore, USP47 knockdown inhibited the migration of A549 cells by blocking the HGF-MET signal pathway (Buus et al., 2009).

Ubiquitin-specific peptidase 47 also participated in the regulation of cell viability and chemotherapeutic resistance in gastric cancer cells (Zhang et al., 2015; Naghavi et al., 2018). USP47 knockdown promoted drug sensitivity in the camptothecin (CPT)- and etoposide (Eto)-resistant gastric cell line NCI-N87 rather than AGS, which is sensitive to CPT and Eto (Naghavi et al., 2018). Mechanically, USP47 was responsible for the activation of NF-κB signaling pathway by promoting the nuclear translocation of RelA. Because CPT and Eto are known to promote NF-κB-dependent apoptosis resistance, USP47 inhibition may be a suitable strategy to overcome drug resistance induces by NF-κB signaling pathway in gastric cancer (Naghavi et al., 2018).

Ubiquitin-specific peptidase 47 is also a potential target for overcoming TKI resistance in treating chronic myelogenous leukemia (CML). Among all DUBs, USP47 showed the highest upregulation in CML cells compared with normal bone marrow (BM) CD34+ cells. Downregulation of USP47 inhibited the proliferation of imatinib-sensitive or drug-resistant CML cells. USP47 knockout significantly inhibited BCR-ABL and BCR-ABL^T315I^-induced mouse CML with reduced of LIN^–^Sca1^+^ c-kit^+^ CML stem cells/progenitor cells. Mechanistic studies have shown that stable Y-box binding protein 1 (YB-1) contributed to USP47-mediated DNA damage repair in CML cells. Inhibiting USP47 *via* the compound P22077 was cytotoxic to CML cells with or without TKI resistance. Additionally, P22077 eliminated leukemia stem/progenitor cells in CML mice while exhibiting low toxicity to mononuclear cells in normal peripheral blood. Therefore, targeting USP47 is an effective and secure strategy to overcome TKI resistance and eradicate leukemia stem/progenitor cells (Lei et al., 2021).

Altogether, USP47 is a promising candidate for cancer targeting. Thus, it is necessary to clarify the mechanisms of the regulation of USP47 expression in cancers.

## Regulation of Ubiquitin-Specific Peptidase 47 Expression in Cancer

Ubiquitin-specific peptidase 47 expression is elevated in many tumors and is involved in multiple expression regulating mechanisms, including transcriptional, post-transcriptional, and post-translational modifications.

### Transcriptional Regulation

There is only one study describing the transcriptional regulation of USP47. USP47 was transcriptionally activated under hypoxia when the transcription factor SOX-9 binds to the USP47 promoter region at −877 to −831 bp, thus promoting its transcription (Choi et al., 2017).

### Post-transcriptional Level

At the post-transcriptional level, many non-coding RNAs are involved in regulating USP47 expression. USP47 is the target protein of miR-204-5p, and high miR-204-5p expression leads to down-regulation of USP47 expression, thereby inhibiting proliferation of gastric cancer and ovarian cancer cells (Zhang et al., 2015; Hu et al., 2020). USP47 is also a target protein of miR-188-5p in CRC. LINC00668 plays an oncogenic role in CRC cells by sponging miR-1885p and upregulating USP47. Overexpression of USP47 could reduce the tumor inhibition induced by LINC00668 knockout (Hu et al., 2019). USP47 is also a target gene of miR-199b, and the miR-199b/USP47/MAPK axis regulates CRC progression (Yan et al., 2019). In osteosarcoma cells, USP47 is the target gene of miR-101-3p, and lncRNA DSCAM-AS1 controls USP47 expression and regulates osteosarcoma progression by binding to miR-101-3p (Guo et al., 2020). In nasopharyngeal carcinoma cells, USP47 is the target gene of miR-454, and lncRNA KCNQ1OT1 regulates USP47 expression by binding to miR-454, thereby promoting cisplatin resistance (Zhang et al., 2020).

### Post-translational Level

At the post-translational level, studies have shown that USP47 interacts with β-TrCP proteins in the E3 ubiquitin ligase complex SCF. USP47 is highly stable. At first, studies showed that β-TrCP could not regulate USP47 expression levels, nor could USP47 regulate β-TrCP enzyme activity (Peschiaroli et al., 2010). Additionally, various stress factors did not change USP47 expression levels. However, later work found that β-TrCP could interact with USP47 through a novel motif “DEGxxxE” in HEK293T cells, and β-TrCP promotes the ubiquitination and degradation of USP47-inactivating mutants rather than wild-type USP47 due to its own ubiquitination properties, possibly explaining the controversial results presented in different studies (Shi et al., 2015). Moreover, endoplasmic reticulum aminopeptidase 1, a key regulator of innate and adaptive anti-tumor immune responses, was found to interact with USP47 and thus interfered with the interaction of USP47 and β-TrCP, leading to ubiquitination degradation of β-TrCP (Bufalieri et al., 2019). These results indicated that USP47 and β-TrCP have a mutual regulation effect, and the expression of USP47 in cells is relatively stable due to its self-deubiquitination characteristic. Therefore, discovering the physiological conditions that regulate the degradation of USP47 protein and the signaling pathways that inhibit its own deubiquitinating enzyme activity is critical to understanding the regulation of USP47. A recent study on CML has shown that the expression of USP47 at the mRNA and protein levels was upregulated in BCR-ABL-overexpressed myeloid progenitor 32D cells. And imatinib (IM) treatment attenuated the expression of USP47. In addition, the RAS or ERK inhibition and STAT5 silencing could also downregulate the BCR-ABL-induced upregulation of USP47 in CML (Lei et al., 2021). Thus, investigating the modulation of USP47 at the mRNA and protein levels, respectively, in these conditions may be helpful to get a complete understanding of USP47.

## Other Pathological Functions of Ubiquitin-Specific Peptidase 47

After introducing the physiological function of USP47 and its role in cancers, we will also introduce the role of USP47 in other diseases.

### Inflammation

The ubiquitin system is involved in inflammation, as the deubiquitinases A20, BRCC3, and USP50 (Lopez-Castejon and Edelmann, 2016). USP47 has been proven to be related to the nuclear localization of NF-κB (Hu et al., 2020). When the canonical NF-κB pathway was stimulated, IκBα was phosphorylated, promoting its β-TrCP-mediated ubiquitinylation and degradation, thereby releasing NF-κB into nucleus. As mentioned above, knockdown of USP47 reduced β-TrCP level and impaired RelA nuclear translocation (Naghavi et al., 2018). Another study showed that USP47 bound to β-TrCP and mediated its deubiquitination to stabilize its expression (Bufalieri et al., 2019). These studies indicated that USP47 plays an important role in modulating the NF-κB signaling pathway, suggesting a role for USP47 in inflammation. Actually, a study has demonstrated the effect of USP47 on the inflammasome activation. The study showed that treatment with P22077, an inhibitor of both USP7 and USP47, impairs NLRP3 inflammasome activation in macrophages, leading to the inhibition of IL-1β, IL-18, and caspase-1 cleavage and release, as well as pyroptotic cell death. Eliminating USP7 and USP47 expression *via* CRISPR-Cas9 in THP-1 macrophages inhibited inflammasome activity, and knocking out USP47 resulted in a more efficient inhibition than did knocking out USP7. A mechanistic study showed that inhibiting USP7 and USP47 led to an increase in the total and K-63 linked NLRP3 ubiquitination and an accumulation of aberrant NLPR3-oligomers. However, whether NLRP3 ubiquitination leads to aberrant NLRP3 oligomers is still unknown. In addition, the USP47 level and activity were modulated by different donors. Although LPS treatment did not induce any differences in either protein level and activity of USP47, nigericin increased both the USP47 level and activity, and an unknown post-translational modification of USP47 may contribute to the modulation induced by nigericin (Palazón-Riquelme et al., 2018). In addition, one study found that knocking down USP47 blocked the passage of the influenza virus into cells (Tran et al., 2013). These findings suggest that USP7 and USP47 are potential therapeutic targets for inflammatory diseases. These findings also enrich our understanding of the role of deubiquitinases in inflammatory responses.

### Myocardial Infarction

One study investigated the roles and mechanisms of members of the USP family in myocardial infarction. Monocytes were isolated from the peripheral blood from patients with myocardial infarction and healthy controls to detect the expressions of multiple USP family members and NF-κB. USP47 and NF-κB expressions were significantly increased during myocardial infarction, and the levels of USP47 and NF-κB were positively correlated. USP47 expression was increased time-dependently in rat H9C2 myocardial ischemia/reperfusion injury model. Downregulation of USP47 decreased apoptosis induced by ischemia/reperfusion injury, increased survivin and cytoplasmic NF-κB, and decreased cleaved caspase-3 and nuclear NF-κB. NF-κB inhibitors could effectively inhibit USP47-induced apoptosis, increase cytoplasmic NF-κB, and decrease nuclear NF-κB. Luciferase reporter genes showed that USP47 promoted NF-κB promoter activity. Therefore, USP47 expression may be related to the progression of myocardial infarction. Downregulating USP47 reduced myocardial ischemia-reperfusion injury by inhibiting apoptosis (Hu et al., 2020).

## Targeting Ubiquitin-Specific Peptidase 47

Owing to the critical role of deubiquitinases in cancer and other diseases, targeted inhibitors are being developed. However, no specific USP47 drugs have been reported yet. Due to the 48.4% similarity between USP47 and USP7 catalytic sites, most inhibitors targeting USP7 also inhibit USP47 activity. *In vitro* assays (e.g., the Ub-CHOP reporter system) and other strategies enabled the discovery of some USP7 inhibitors, including PR-619, P22077, and HBX 41,108 (Colland et al., 2009; Altun et al., 2011; Gavory et al., 2018; Engström et al., 2020). PR-619 is a cell-permeable, pyridine broad-spectrum deubiquitinase inhibitor, targeting ATXN3; BAP1; JOSD2; OTUD5; UCH-L1; UCH-L3; UCH-L5/UCH37; USP1, 2, 4, 5, 7, 8, 9X, 10, 14, 15, 16, 19, 20, 22, 24, 28, 47, 48; VCIP135; and YOD1. The other inhibitor, P22077[1-(5-((2,4-difluorophenyl)thio)-4-nitrothiophen-2-yl)ethanone], selectively inhibits the activities of USP7 and USP47 (Altun et al., 2011). Therefore, some studies used PR619 and P22077 as functional research tools for USP7 and USP47 (Setz et al., 2017). Additionally, one study focused on developing dual small-molecule enzyme inhibitors, named compound 1–14, targeting USP7 and USP47. The main advantage of dual inhibitors is their ability to inhibit multiple signaling pathways simultaneously, thus enhancing the actions of anti-cancer drugs and reducing the generation of drug resistance (Weinstock et al., 2012). Specific USP7 inhibitors have been developed, such as FT671, which targets a unique dynamic pocket near the catalytic center of the auto-inhibited apo-form of USP7 (Turnbull et al., 2017).

Analyzing the differences between USP47 and USP7 will aid the development of USP47-specific inhibitors. First, although USP47 shares a high similarity with USP7 in the sequences of their catalytic core, their differences are enough to cause different affinities to compounds. A study of USP7-specific inhibitors showed that six residues of the compound binding site are different from USP7 (Gavory et al., 2018). And the sequence alignment of the catalytic cores of USP7, USP40, and USP47 was performed by MUSCLE. The results showed a specific sequence of USP47 at 431–485aa, which may be a potential target of USP47 (Supplementary Figure 1). Second, the tertiary structure of USP47 also differed from USP7. The structure study of FT671 and FT827 targeting USP7 found that USP7 had a unique switching loop at 283–295aa, leading to unique positions of Tyr465 and Tyr514 in the ubiquitin-binding channel and allowing FT671 and FT827 to specifically target USP7 (Turnbull et al., 2017). Finally, USP47 could bind to its substrate through a specific motif. For example, USP47 could bind to β-TrCP through its unique “**DEG**ICL**E**” motif (Shi et al., 2015). The distinct substrate-specific binding sites on USP47 provide attractive targets for potential therapeutic intervention by manipulating USP47 activity toward a specific substrate. Altogether, these showed that USP47 and USP7 are different enough to allow USP47-specific targeting. Research on the structure of USP47 will be of great benefit to develop specific USP47 inhibitors.

## Conclusion

Deubiquitination is a vital protein modification involved in cell proliferation, cell differentiation, apoptosis, body development, and disease occurrence. The USP47 structure and function data highlight that USP47 is a critical regulator of neurological diseases, cancers, and other diseases. USP47 is overexpressed in multiple cancers, such as CRC and breast cancer, and it may be a valuable biomarker for diagnosis and prognosis (Pan et al., 2020; Silvestrini et al., 2020).

**FIGURE 2:**
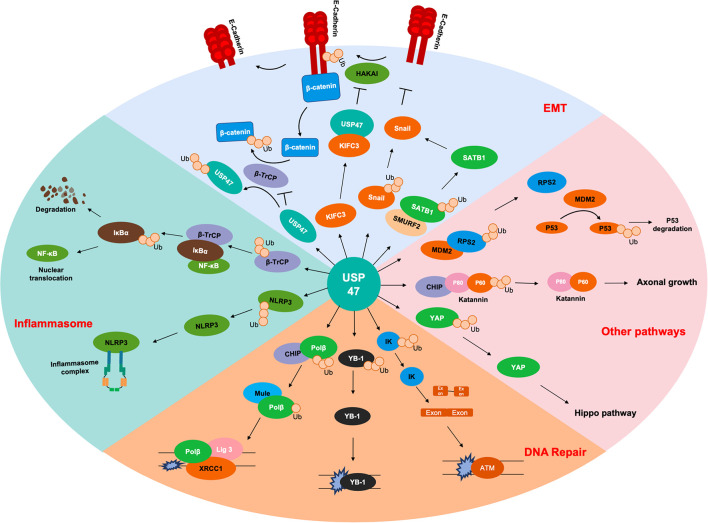
Substrates of USP47. Several proteins are deubiquitinated by USP47 during EMT, including SATB1, Snail, and β-catenin. USP47 is essential for maintaining epithelial cell adhesion *via* E-cadherin deubiquitination. USP47 promotes the nuclear translocation of NF-κB by stabilizing β-TrCP, which is the ubiquitinase of IκBα. USP47 also facilitates the formation of inflammasome complex, which may be due to the deubiquitination of NLRP3. USP47 promotes the DNA repair process through deubiquitinating Polβ, YB-1, and IK. In addition, USP47 also modulates the P53 and Hippo signaling pathways through deubiquitinating RPS2 and YAP, respectively. Finally, the deubiquitination of Katanin-p60 promotes the axonal growth of hippocampal neurons.

As shown in Table 1 and Figure 2, USP47 modulates several biological processes, including EMT, DNA repair, axonal growth and so on. It is well known that Lys48-linked polyUb chain acts as a signal for degradation by the proteasome and Lys63-linked polyUb chain serves as a scaffold activity signaling transduction cascades (Komander and Rape, 2012). USP47 removed Lys48-linked polyUb chains of DNA polymerase β, Katanin-p60, and E-cadherin and liberated the Ub moiety from Lys63-linked polyUb chains in NLRP3 (Parsons et al., 2011; Yang et al., 2013; Sako-Kubota et al., 2014; Palazón-Riquelme et al., 2018). These indicated that USP47 not only participated in the regulation of degradation but also mediated the signal transduction process. Given that most studies currently focus on the degradation modulation of USP47, it is crucial to further investigate its substrates with Lys63-linked polyUb chains.

**TABLE 1:** The biological functions of USP47.

Interactors	Functional outcomes	References
Katanin	Promoting the Katanin-p60-mediated axonal growth in hippocampal neurons	Yang et al., 2013
KIFC3	Maintaining stable cell-cell adhesion by suppressing the E-cadherin degradation	Sako-Kubota et al., 2014
Polβ	Base excised repair pathway, repair damage DNA	Weinstock et al., 2012
IK	Increasing the level of ATM through IK deubiquitination	Ka et al., 2020
β-TrCP	Significance unclear; promotes USP47 ubiquitination; inhibit β-TrCP degradation, modulation of Gli transcription factors	Peschiaroli et al., 2010; Shi et al., 2015; Bufalieri et al., 2019
YAP	Stabilization of YAP contributes to the progression of colorectal cancer	Pan et al., 2020
Snail	Promoting EMT	Choi et al., 2017
SATB1	Promoting colorectal cancer proliferation, migration, tumorigenesis, and resistance to 5-FU	Yu et al., 2019
RPS2	Inhibiting the interaction between RPS2 and MDM2, promoting P53 wild-type colorectal cancer proliferation and clone formation	Cho et al., 2020
β-catenin	Activating Wnt signaling pathway	Shi et al., 2015
YB-1	DNA damage repair	Lei et al., 2021
NLRP3	Regulating the activity of inflammatory bodies in macrophages	Palazón-Riquelme et al., 2018

The expression of proteins is regulated at the transcriptional, post-transcriptional, and post-translational levels. It has been confirmed that SOX-9 is responsible for regulating the transcription of USP47, while a variety of non-coding RNAs were involved in regulating the post-transcription level of USP47 under different physiological conditions. The protein level of USP47 is maintained through its own deubiquitination (Shi et al., 2015; Choi et al., 2017; Yan et al., 2019). Therefore, the regulation of USP47 expression may mainly focus on the transcriptional level and post-transcriptional level. Finding the post-transcription modifications that inhibit the activity of USP47 deubiquitinase is also essential.

Besides, although USP47 has been proved to have a conserved catalytic core region in its amino acid sequence, no USP47 crystal structure has been reported. This largely limits the discovery of the unique structure of USP47 that is different from USP7 and USP40 and also limits the development of USP47-specific inhibitors. In addition, USP47 plays diverse roles in cancers and myocardial infarction by regulating various substrates. The side-effects of USP47 inhibitors may be unavoidable. Therefore, the delivery of multispecific drugs that enrich the USP47 inhibitors accumulated at a particular site through some tissue-specific markers would be of great help (Deshaies, 2020), and there is an urgent need to clarify the specific motif mediating the binding of a specific substrate with USP47, such as the “DEGxxxE” motif-mediated β-TrCP binding. Targeting such a specific motif of USP47 may be a great strategy to treat some specific diseases.

According to the effect of USP47 in the regulation of axonal growth in hippocampal neurons, it is meaningful to investigate the role of USP47 in neurodegenerative disease and neurodevelopment (Yang et al., 2013). Additionally, a conditional USP47 knockout or overexpression mouse model will help investigate the role of USP47 in development and diseases.

This mini review summarized the physiological and pathological functions of the deubiquitinase, USP47, as well as the development of potential targeted inhibitors against this enzyme. This review will provide a basis for studying the mechanisms of USP47 and developing therapeutic strategies for cancers and other diseases.

## Author Contributions

KP: substantial contributions to the conception of this work and revising this work critically for important intellectual content. JF: contributions to the acquisition of data for this work. WX: substantial contributions to the design of this work and drafting this work. All authors approved the version to be published and agreed to be accountable for all aspects of this work.

## Conflict of Interest

The authors declare that the research was conducted in the absence of any commercial or financial relationships that could be construed as a potential conflict of interest.

## Publisher’s Note

All claims expressed in this article are solely those of the authors and do not necessarily represent those of their affiliated organizations, or those of the publisher, the editors and the reviewers. Any product that may be evaluated in this article, or claim that may be made by its manufacturer, is not guaranteed or endorsed by the publisher.

## Footnotes

1
https://www.genecards.org/cgi-bin/carddisp.pl?gene=USP47#summaries

2
https://www.proteinatlas.org/ENSG00000170242-USP47
