# Supplementary figures and images for: Role of Ubiquitin-Specific Peptidase 47 in Cancers and Other Diseases
Source: Front Cell Dev Biol. 2021 Sep 17;9:726632. doi: 10.3389/fcell.2021.726632 (PMC8484750; doi:10.3389/fcell.2021.726632)

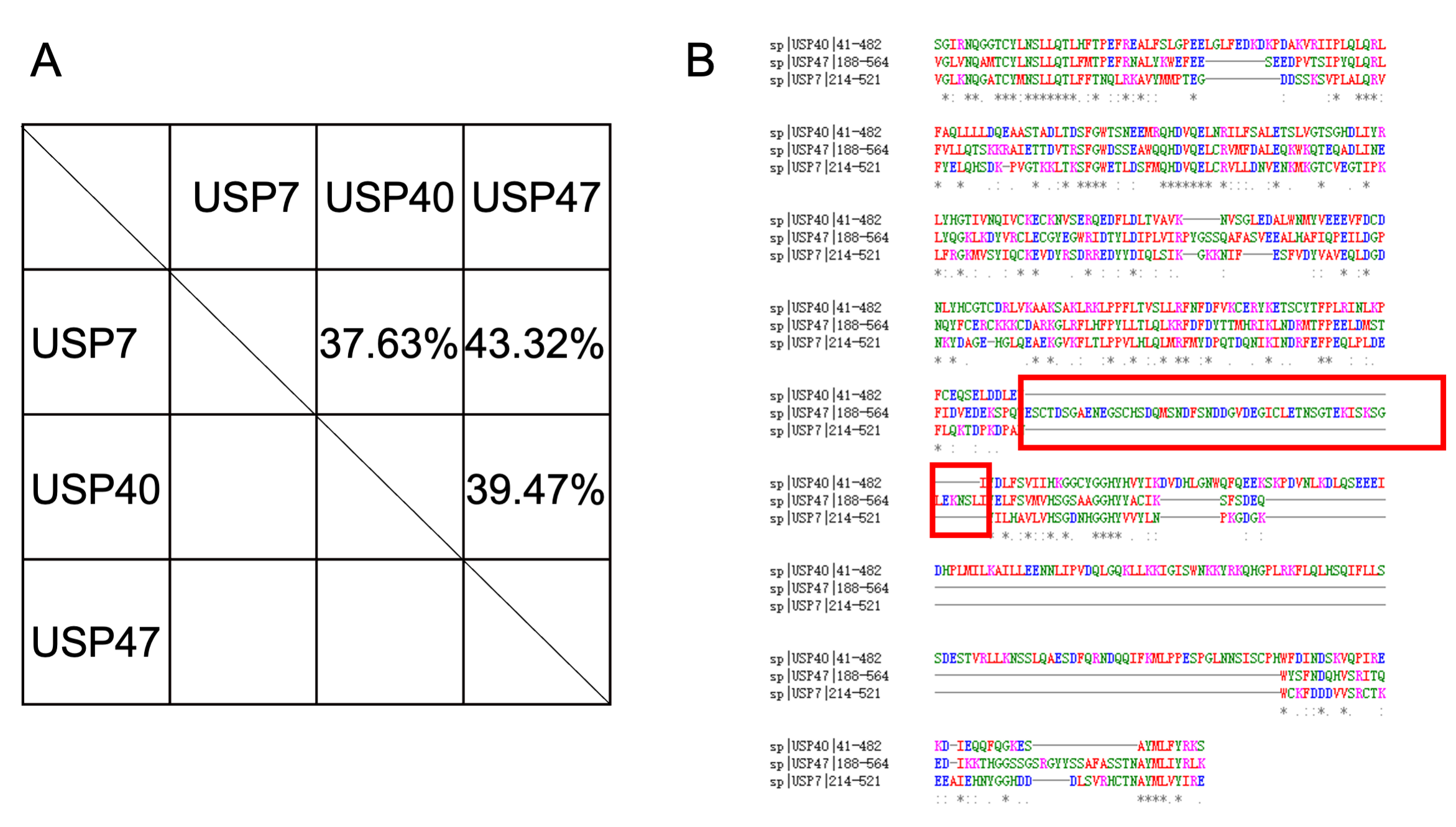

Supplement: Supplementary file 1 [file Image_1.TIFF]
