# Supplementary material for: Role of Ubiquitin-Specific Peptidase 47 in Cancers and Other Diseases
Source: Front Cell Dev Biol. 2021 Sep 17;9:726632. doi: 10.3389/fcell.2021.726632 (PMC8484750; doi:10.3389/fcell.2021.726632)
Supplement: Supplementary file 2 [file Data_Sheet_1.docx]

**Figure S1. Sequence similarity between DUBs.** (A) Percentages of amino acid sequence identity within catalytic core domain among the four DUBs was determined using the MUSCLE program (https://www.ebi.ac.uk/Tools/msa/muscle/). The sequence data used for analysis are as follows; human USP7, Q93009; human USP40, Q9NVE5; human USP47, Q96K76. (B) The CLUSTAL multiple sequence alignment by MUSCLE.
